# Clinical significance of Osaka prognostic score based on nutritional and inflammatory status in patients with esophageal squamous cell carcinoma

**DOI:** 10.1186/s12885-022-09406-6

**Published:** 2022-03-17

**Authors:** Jifeng Feng, Lifen Wang, Liang Wang, Xun Yang, Guangyuan Lou

**Affiliations:** 1 Department of Thoracic Oncological Surgery, Institute of Cancer Research and Basic Medical Sciences of Chinese Academy of Sciences, Cancer Hospital of University of Chinese Academy of Sciences, Zhejiang Cancer Hospital, Hangzhou, 310022 China; 2 Department of Operating Theatre, Nursing Department, Institute of Cancer Research and Basic Medical Sciences of Chinese Academy of Sciences, Cancer Hospital of University of Chinese Academy of Sciences, Zhejiang Cancer Hospital, Hangzhou, 310022 China; 3 Department of Medical Oncology, Institute of Cancer Research and Basic Medical Sciences of Chinese Academy of Sciences, Cancer Hospital of University of Chinese Academy of Sciences, Zhejiang Cancer Hospital, Hangzhou, 310022 China

**Keywords:** Esophageal squamous cell carcinoma, Osaka prognostic score, Cancer-specific survival, C-reactive protein, Albumin, Total lymphocyte count

## Abstract

**Background:**

It has been reported that Osaka prognostic score (OPS), based on C-reactive protein (CRP), total lymphocyte counts (TLC) and albumin (ALB), was relevant to prognosis in colorectal cancer. However, the role of OPS regarding prognosis in patients with esophageal squamous cell carcinoma (ESCC) has not been reported. The current study aimed to explore the clinical outcome of OPS and establish and validate a nomogram for survival prediction in ESCC after radical resection.

**Methods:**

This retrospective study included 395 consecutive ESCC patients with radical resection. Then patients were randomly divided into two cohorts: training cohort (276) and validation cohort (119). The OPS, based on TLC, CRP and ALB, was constructed to verify the prognostic value by Kaplan-Meier curves and Cox analyses. A nomogram model for prognosis prediction of cancer-specific survival (CSS) was developed and validated in two cohorts.

**Results:**

Kaplan-Meier curves regarding the 5-year CSS for the groups of OPS 0, 1, 2 and 3 were 55.3, 30.6, 17.3 and 6.7% (*P* < 0.001) in the training cohort and 52.6, 33.3, 15.8 and 9.1% (*P* < 0.001) in the validation cohort, respectively. Then the OPS score in multivariate Cox analysis was confirmed to be a useful independent score. Finally, a predictive OPS-based nomogram was developed and validated with a C-index of 0.68 in the training cohort and 0.67 in the validation cohort, respectively. All above results indicated that the OPS-based nomogram can accurately and effectively predict survival in ESCC after radical resection.

**Conclusion:**

The OPS serves as a novel, convenient and effective predictor in ESCC after radical resection. The OPS-based nomogram has potential independent prognostic value, which can accurately and effectively predict individual CSS in ESCC after radical resection.

## Introduction

Global cancer statistics 2018 revealed that esophageal cancer (EC) is one of the most common cancers worldwide with a total of 0.57 million new cases diagnosed and 0.51 million cases died from cancer [1]. Esophageal squamous cell carcinoma (ESCC) accounts for the majority of patients with EC, particularly in the high-incidence regions of China [2]. Despite advances in diagnosis and treatment in recent years, the survival prognosis for ESCC remains not satisfactory, mainly because the majority patients are diagnosed at advanced stages and lose the probability of curative resection [2, 3]. Therefore, the late diagnosis and poor prognosis of ESCC highlights the need to refine more sensitive and effective prediction methods, which are essential prior to treatment.

A growing number of studies revealed that cancer progression and prognosis is associated with nutritional and inflammatory status [4, 5]. Therefore, various inflammatory and/or nutritional indicators have been applied either alone or in combination to cancers in recent years. Serum C-reactive protein (CRP) and albumin (ALB) were the most widely recognized indicators to predict prognosis in a variety of cancers, including ESCC [6, 7]. The score system of Glasgow prognostic score (GPS) based on ALB and CRP was also confirmed as one of the most widely recognized scores for predicting clinical outcomes in a variety of cancers [8–10]. Moreover, a series of other indexes about inflammation and/or nutrition, such as prognostic nutritional index (PNI), systemic immune-inflammation index (SII) and systemic inflammation score (SIS), have also been confirmed to be associated with tumor prognosis [11–14].

Recently, a novel prognostic score based on the inflammatory and nutritional predictors, named Osaka Prognostic Score (OPS), was proposed for the first time to predict the prognosis in colorectal cancer (CRC) after radical resection [15]. Compared with other prognostic scores, the results demonstrated that the OPS, based on serum CRP, ALB and total lymphocyte count (TLC), had a reliable ability to predict prognosis in 511 CRC patients with radical resection. However, the application of OPS needs to be confirmed in other cancers. To date, moreover, there have been no reports regarding OPS in ESCC. Therefore, we initially explored the significance of OPS in patients with ESCC after radical resection for predicting cancer-specific survival (CSS). Finally, a nomogram based on OPS was also constructed and validated to predict individual survival for patients in ESCC after radical resection.

## Materials and methods

### Ethical statement

This study was approved by the ethics committee of Zhejiang Cancer Hospital (IRB.2021–6) and was performed in accordance with the Declaration of Helsinki. All retrospective data including in this study was anonymous, therefore, informed consent was waived by the ethics committee of Zhejiang Cancer Hospital.

### Study population

**Fig. 1:**
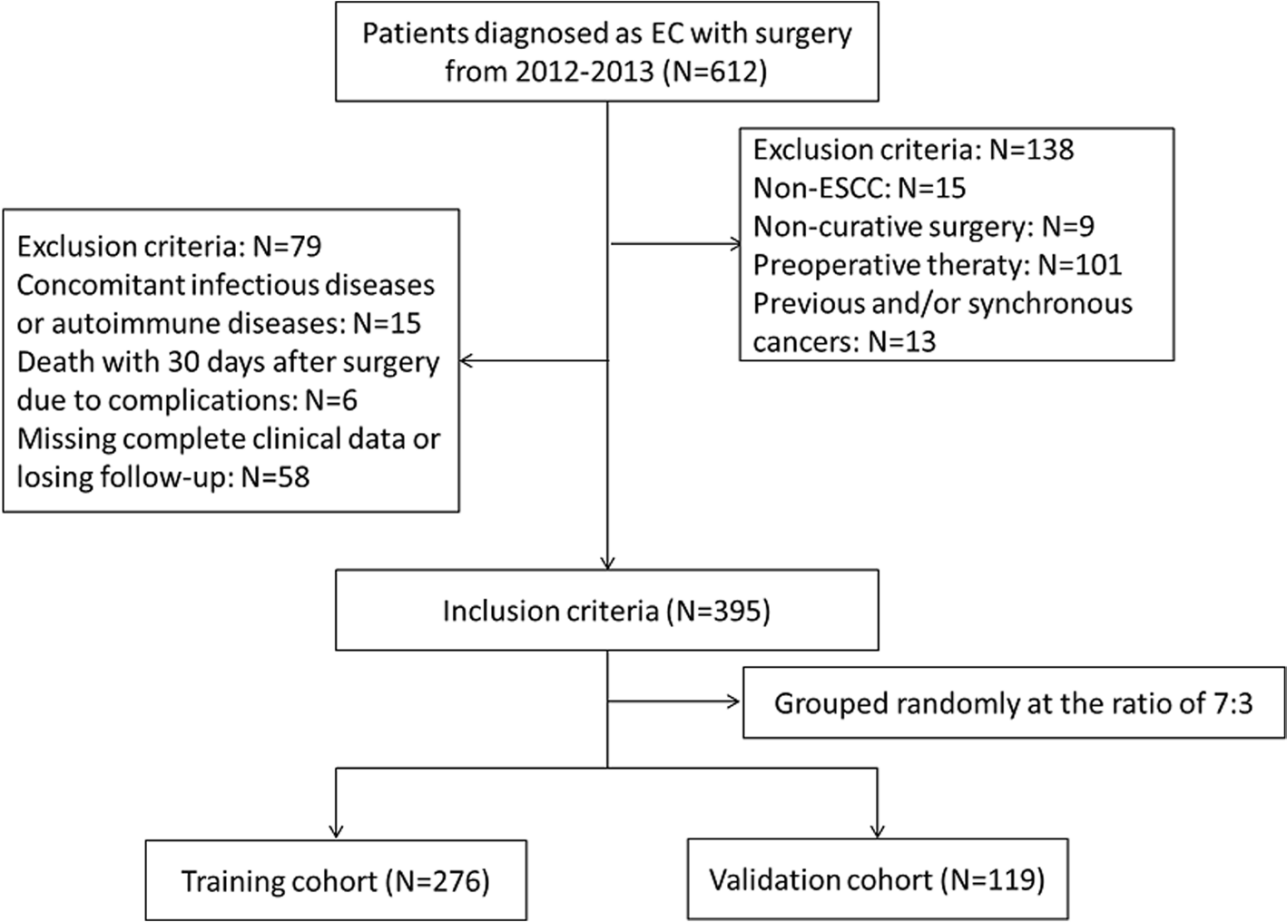
The flow diagram of selection of eligible patients. According to the inclusion and exclusion criteria, a total of 395 patients were randomly divided into either a training cohort (*n* = 276) or validation cohort (*n* = 119) at a ratio of 7:3 for further analysis

Between 2012 and 2013, a total of 612 consecutive patients with EC with surgery in our department were retrospectively collected and analyzed. Patients who did not undergo radical resection and/or had any missing clinical or laboratory information were excluded from the study. The detail inclusion and exclusion criteria were shown in Fig. 1. Finally, the clinical records of the remaining 395 patients, who underwent above radical resection for ESCC, were retrospectively reviewed. All patients were then randomly assigned to a training cohort (*n* = 276) or validation cohort (*n* = 119) at a ratio of 7:3.

### Treatment and follow-up

All patients underwent radical resection in the current study. The radical resection included the Ivor Lewis or McKeown procedure with two-field lymphadenectomy [16, 17]. The 8th AJCC/UICC TNM staging system was carried out for the current study [18]. Postoperative adjuvant treatment was still uncertain at that time. NCCN guidelines only recommend regular follow-up for those patients after radical resection. Thus, not all ESCC patients in China have received postoperative adjuvant therapy, which is mainly performed according to the postoperative pathological results as well as the physical and financial status of each patient [19, 20]. According to the previous studies, postoperative adjuvant treatments were carried out including cisplatin-based chemotherapy and/or radiotherapy, but not mandatory, for ESCC patients with positive lymph node metastasis and those with T3-T4 stage [21, 22]. Patients typically received a median of 4 cycles of postoperative chemotherapy consisting of cisplatin with fluorouracil or paclitaxel/docetaxel. Postoperative radiotherapy was consisted of three-dimensional conformal radiotherapy (3D-CRT) or intensity-modulated radiotherapy (IMRT), which was initiated 4–8 weeks after radical resection with a median dosage of 50 Gy (1.8–2 Gy/fraction and 5 fractions per week) [19, 21, 22]. The patients were followed up with regular checks in our outpatient department. The routine examination items included physical examination, laboratory tests, tumor markers, thoracic CT scanning and esophageal barium. The last follow-up was completed in Dec. 2019.

### Data collection and OPS definition

**Fig. 2:**
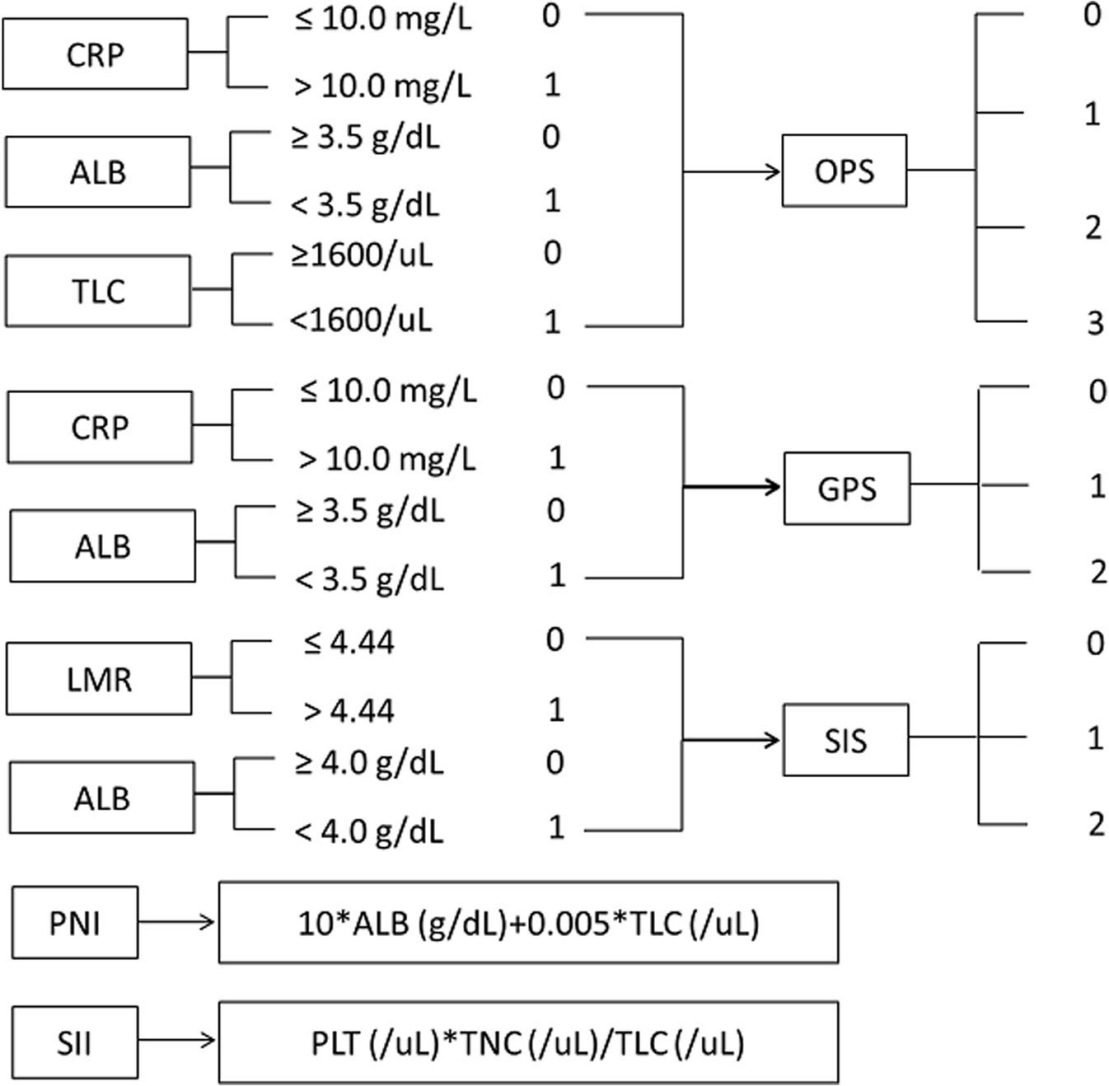
Calculation of the inflammatory and/or nutritional scores. The OPS based on CRP, ALB and TLC calculated into 4 groups. The GPS based on CRP and ALB calculated into 3 groups. The SIS based on ALB and LMR calculated into 3 groups. The PNI based on ALB and TLC calculated into 2 groups. The SII based on PLT, TNC and TLC calculated into 2 groups

The clinical data including age, gender, tumor location, tumor length, differentiation, vessel invasion, perineural invasion and TNM stage and laboratory results including serum CRP, ALB, TLC, platelet (PLT), total neutrophil count (TNC) and total monocyte count (TMC) were retrospectively collected from our medical records. The above laboratory results were obtained within 1 week before surgery. The definitions of SIS, SII, PNI and GPS refer to the previous studies [11–14]. The OPS was calculated by the following three variables: CRP (≤ 10.0 mg/L: 0 point and > 10.0 mg/L: 1 point), ALB (≥ 3.5 g/dL: 0 point and < 3.5 g/dL: 1 point) and TLC (≥1600/uL: 0 point and < 1600/uL: 1 point). The OPS then was calculated as the summed score of 0 or 1, which divided into 4 groups. The detailed calculations of OPS, GPS, SIS, PNI and SII were shown in Fig. 2.

### Statistical analysis

Medcalc 17.6 (MedCalc Software bvba, Ostend, Belgium), R software (version 3.6.1, Vienna, Austria) and SPSS 20.0 (SPSS Inc., Chicago, IL, USA) were used to perform all statistical analyses in the current study. The areas under the curve (AUC) between OPS and other variables (SIS, SII, PNI and GPS) were compared by receiver operating characteristic (ROC) curves. The Kaplan-Meier method was used to compare the CSS. Cox regression analyses were performed to confirm independent factors. A prognostic nomogram was build based on the results in multivariate analyses. Calibrations of for survival prediction were performed by comparing the two cohorts. Time-dependent ROC curves and decision curves were also performed to evaluate the discriminative ability and predictive accuracy. All statistical tests were two-side and a *P* value < 0.05 was considered to be statistically significant.

## Results

### Patient characteristics in two cohorts

The baseline characteristics between the two cohorts were shown in Table 1. The median follow-up time was 39 months (range 9–92 months) in the training cohort and 42 months (range 7–90 months) in the validation cohort, respectively. Based on the criteria of the 8th edition AJCC TNM staging system, there were 79 (28.6%), 94 (34.1%) and 103 (37.3%) cases in stage I, II, and III in the training cohort and 33 (27.7%), 46 (38.7%) and 40 (33.6%) cases in the validation cohort, respectively. There were more male patients in the validation cohort than those in the training cohort (79.0% vs. 68.1%, *P* = 0.028). Otherwise, there was no significance difference between the two groups.

**Table 1:** Baseline characteristics of ESCC patients in the training and validation sets

	Training set (*n* = 276, %)	Validation set (*n* = 119, %)	*P* value
Age (mean ± SD, years)	59.0 ± 7.9	57.9 ± 7.7	0.177
Gender (male/female)	188(68.1)/88(31.9)	94(79.0)/25(21.0)	0.028
Tumor length (mean ± SD, cm)	4.2 ± 1.8	4 .3 ± 1.7	0.761
Tumor location (upper/middle/lower)	17(6.2)/122(44.2)/137(49.6)	10(8.4)/54(45.4)/55(46.2)	0.658
Vessel invasion (no/yes)	231(83.7)/45(16.3)	99(83.2)/20(16.8)	0.902
Perineural invasion (no/yes)	221(80.1)/55(19.9)	97(81.5)/22(18.5)	0.740
Differentiation (well/moderate/poor)	41(14.9)/184(66.7)/51(18.4)	17(14.3)/78(65.5)/24(20.2)	0.924
TNM stage (I/II/III)	79(28.6)/94(34.1)/103(37.3)	33(27.7)/46(38.7)/40(33.6)	0.659
Adjuvant treatment (no/yes)	198(71.7)/78(28.3)	87(73.1)/32(26.9)	0.780
CRP (mean ± SD, mg/L)	7.2 ± 8.0	7.6 ± 7.9	0.633
ALB (mean ± SD, g/dL)	4.1 ± 0.5	4.0 ± 0.5	0.454
PLT (mean ± SD, 10^9/L)	224 ± 71	225 ± 75	0.935
TNC (mean ± SD, 10^9/L)	4.42 ± 1.54	4.55 ± 1.62	0.471
TLC (mean ± SD, 10^9/L)	1.58 ± 0.5	1.54 ± 0.4	0.395
TMC (mean ± SD, 10^9/L)	0.52 ± 0.20	0.50 ± 0.13	0.548
PNI (mean ± SD)	48.6 ± 5.5	48.0 ± 6.0	0.297
SII (mean ± SD)	674.9 ± 355.5	720.4 ± 416.8	0.269
OPS (0/1/2/3)	85(30.8)/124(44.9)/52(18.8)/15(5.5)	38(31.9)/51(42.9)/19(16.0)/11(9.2)	0.507
GPS (0/1/2)	182(65.9)/69(25.0)/25(9.1)	78(65.5)/29(24.4)/12(10.1)	0.947
SIS (0/1/2)	140(50.7)/118(42.8)/18(6.5)	63(52.9)/51(42.9)/5(4.2)	0.654

*ESCC* Esophageal squamous cell carcinoma, *SD* Standard deviation, *CRP* C-reactive protein, *ALB* Albumin, *PLT* Platelet, *TNC* Total neutrophil count, *TLC* Total lymphocyte count, *TMC* Total monocyte count, *OPS* Osaka prognostic score, *GPS* Glasgow prognostic score, *SIS* Systemic inflammation score, *TNM* Tumor node metastasis, *PNI* Prognostic nutritional index, *SII* Systemic immune-inflammation index

### Patient characteristics grouped by OPS

The results in the current study demonstrated that OPS was significantly associated with various baseline variables, such as TNM stage, vessel and perineural invasion, tumor length, differentiation, GPS, SIS, PNI and SII. The detailed baseline characteristics grouped by OPS was shown in Table 2.

**Table 2:** Comparison of baseline characteristics of ESCC patients based on OPS in training set

	OPS 0 (85, %)	OPS 1 (124, %)	OPS 2 (52, %)	OPS 3 (15, %)	*P* value
Age (years, ≤60/> 60)	54(63.5)/31(36.5)	74(59.7)/50(40.3)	25(48.1)/27(51.9)	11(73.3)/4(26.7)	0.205
Gender (male/female)	58(68.2)/27(31.8)	81(65.3)/43(34.7)	39(75.0)/13(25.0)	10(66.7)/5(33.3)	0.660
Tumor length (cm, ≤3.0/> 3.0)	34(40.0)/51(60.0)	40(32.3)/84(67.7)	9(17.3)/43(82.7)	1(6.7)/14(93.3)	0.007
Tumor location (upper/middle/lower)	4(4.7)/36(42.4)/45(52.9)	8(6.5)/57(46.0)/59(47.5)	4(7.7)/22(42.3)/26(50.0)	1(6.7)/7(46.7)/7(46.7)	0.984
Vessel invasion (no/yes)	77(90.6)/8(9.4)	103(83.1)/21(16.9)	43(82.7)/9(17.3)	8(53.3)/7(46.7)	0.004
Perineural invasion (no/yes)	76(89.4)/9(10.6)	94(75.8)/30(24.2)	39(75.0)/13(25.0)	10(66.7)/5(33.3)	0.041
Differentiation (well/moderate/poor)	15(17.6)/61(71.8)/9(10.6)	15(12.1)/81(65.3)/28(22.6)	9(17.3)/36(69.2)/7(13.5)	2(13.3)/6(40.0)/7(46.7)	0.025
TNM stage (I/II/III)	25(29.4)/39(45.9)/21(24.7)	45(36.3)/34(27.4)/45(36.3)	8(15.4)/17(32.7)/27(51.9)	1(6.7)/4(26.7)/10(66.7)	< 0.001
Adjuvant treatment (no/yes)	65(76.5)/20(23.5)	84(67.7)/40(32.3)	38(73.1)/14(26.9)	11(73.3)/4(26.7)	0.576
GPS (0/1/2)	85(100)/0(0)/0(0)	97(78.2)/27(21.8)/0(0)	0(0)/42(80.8)/10(19.2)	0(0)/0(0)/15(100)	< 0.001
SIS (0/1/2)	52(61.2)/28(32.9)/5(5.9)	74(59.7)/41(33.1)/9(7.2)	14(26.9)/35(67.3)/3(5.8)	0(0)/14(93.3)/1(6.7)	< 0.001
PNI (≤47.5/> 47.5)	13(15.3)/72(84.7)	58(46.8)/66(53.2)	37(71.2)/15(28.8)	15(100)/0(0)	< 0.001
SII (≤558/> 558)	45(52.9)/40(47.1)	50(40.3)/74(59.7)	22(42.3)/30(57.7)	1(6.7)/14(93.3)	0.008

*ESCC* Esophageal squamous cell carcinoma, *OPS* Osaka prognostic score, *GPS* Glasgow prognostic score, *SIS* Systemic inflammation score, *PNI* Prognostic nutritional index, *SII* Systemic immune-inflammation index, *TNM* Tumor node metastasis

### AUC comparisons between OPS and other variables

**Fig. 3:**
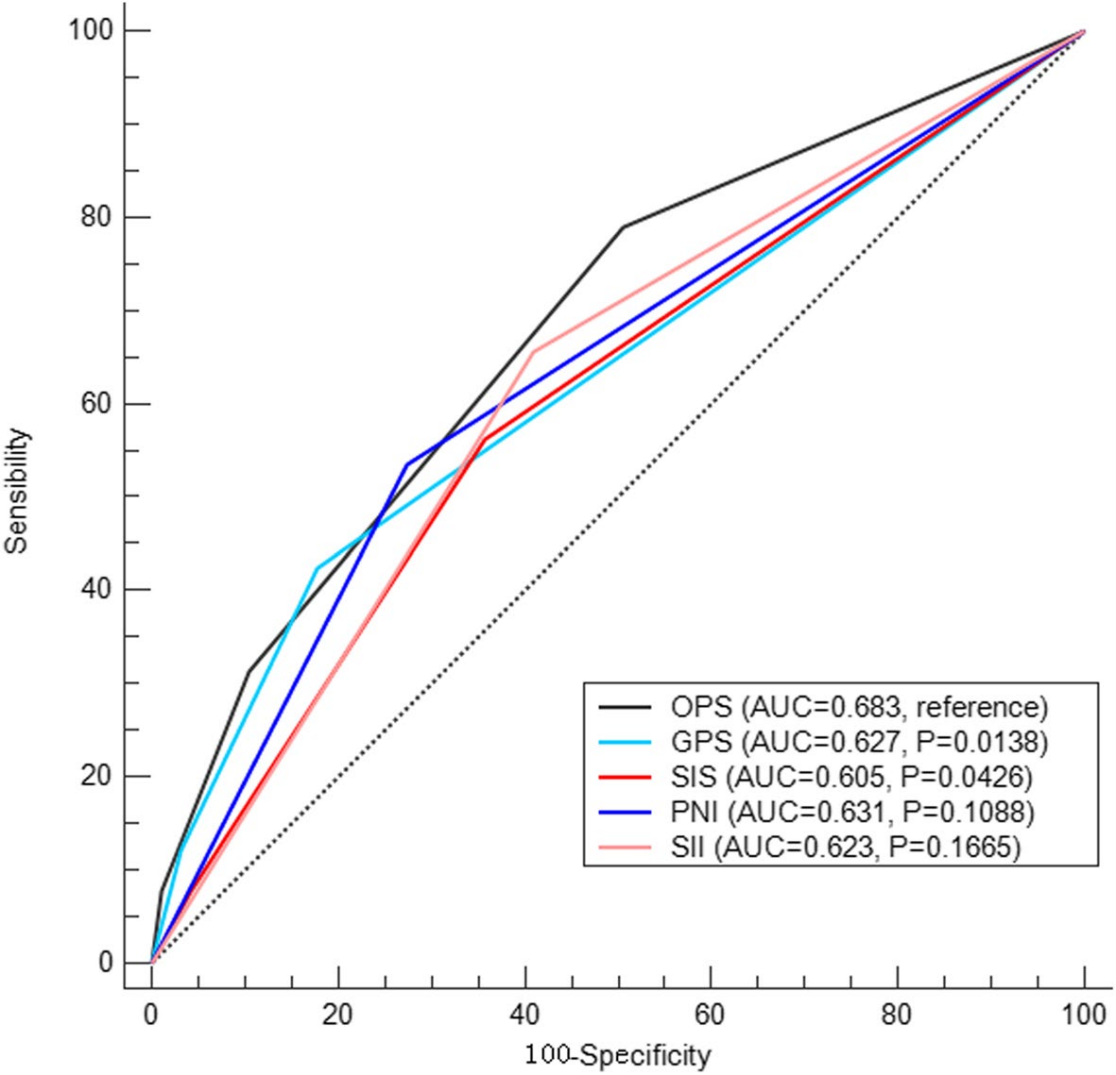
AUC comparisons between OPS and other variables. The results indicated that OPS (AUC = 0.683) had the largest AUC compared with GPS (AUC = 0.627, *P* = 0.0138), SIS (AUC = 0.605, *P* = 0.0426), PNI (AUC = 0.631, *P* = 0.1088) and SII (AUC = 0.623, *P* = 0.1665). The results indicated that higher predictive ability of OPS than other indicators

The AUC values comparisons according to the ROC curves between OPS and other variables (GPS, SIS, PNI and SII) were shown in Fig. 3. The AUC value regarding OPS was 0.683, indicated that OPS had the largest AUC compared with GPS (*P* = 0.0138, AUC = 0.627), SIS (*P* = 0.0426, AUC = 0.605), PNI (*P* = 0.1088, AUC = 0.631) and SII (*P* = 0.1665, AUC = 0.623). These results indicated that higher predictive ability of OPS on prognosis than other indicators.

### CSS analyses and univariate and multivariate analyses

**Fig. 4:**
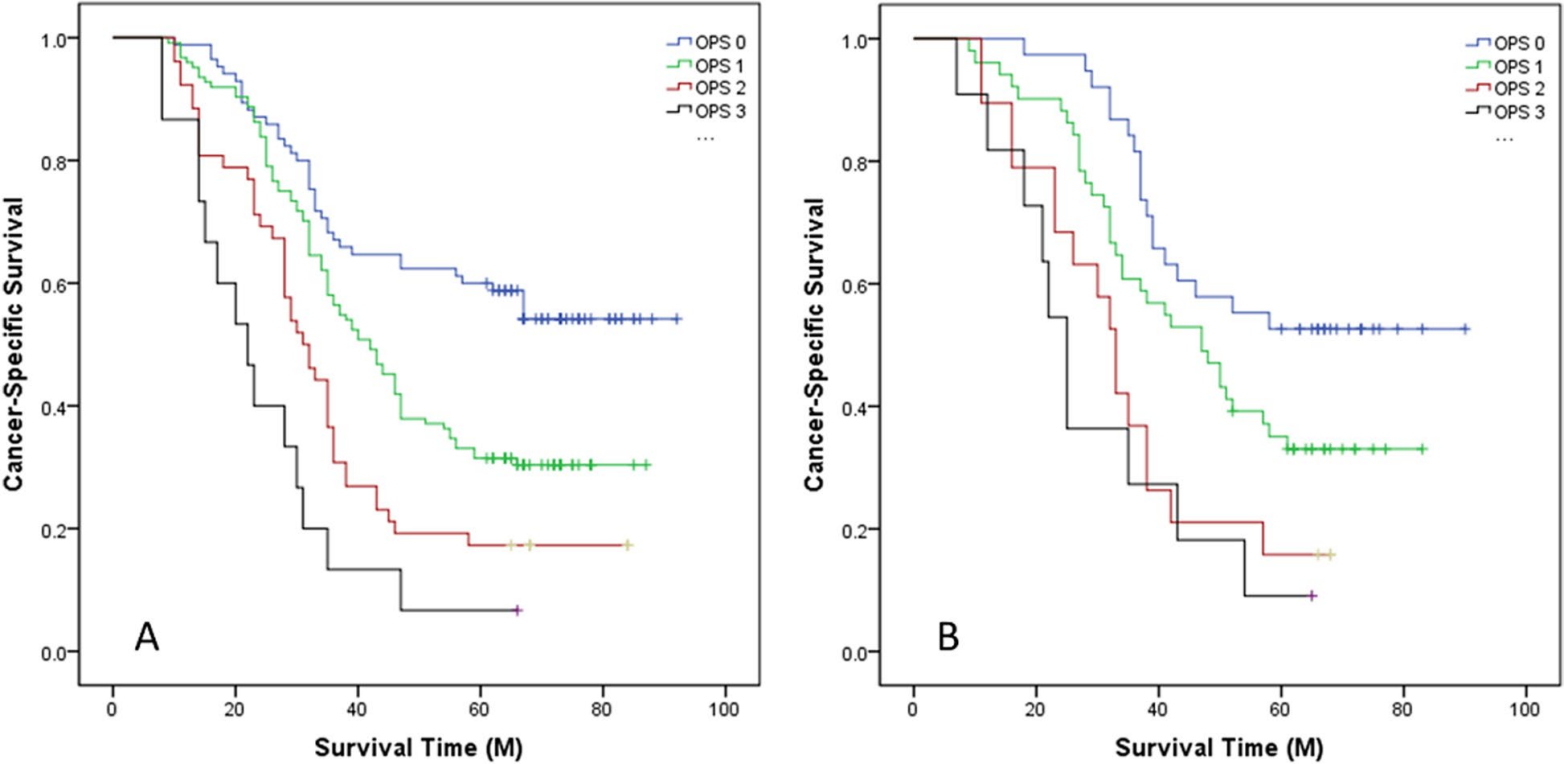
CSS analyses grouped by OPS. Kaplan-Meier curves revealed that 5-year CSS for groups of OPS 0, 1, 2 and 3 were 55.3, 30.6, 17.3 and 6.7% in training cohort (*P* < 0.001, **A**) and 52.6, 33.3, 15.8 and 9.1% in validation cohort (*P* < 0.001, **B**), respectively

The 5-year CSS for the groups of OPS 0, 1, 2 and 3 were 55.3, 30.6, 17.3 and 6.7% in training cohort and 52.6, 33.3, 15.8 and 9.1% in validation cohort, respectively (*P* < 0.001, Fig. 4). The result revealed that OPS confirmed as an independent score associated with CSS according to the multivariate analysis (Table 3).

**Table 3:** Univariate and multivariate Cox analyses of CSS in training set

	Univariate analyses		Multivariate analyses	
	HR (95% CI)	*P* value	HR (95% CI)	*P* value
Age (years, > 60/≤60)	0.925 (0.687–1.245)	0.606		
Gender (male/female)	0.872 (0.640–1.188)	0.386		
Tumor length (cm, > 3.0/≤3.0)	1.266 (0.919–1.745)	0.149		
Tumor location				
middle/upper	1.175 (0.609–2.266)	0.631		
lower/upper	1.149 (0.598–2.209)	0.677		
Vessel invasion (yes/no)	1.657 (1.149–2.388)	0.007		
Perineural invasion (yes/no)	1.564 (1.116–2.193)	0.009		
Differentiation				
moderate/well	1.146 (0.745–1.762)	0.534		
poor/well	1.334 (0.796–2.236)	0.274		
TNM stage				
II/I	1.723 (1.150–2.582)	0.008	1.814 (1.200–2.743)	0.005
III/I	2.555 (1.734–3.765)	< 0.001	1.981 (1.317–2.980)	< 0.001
Adjuvant treatment (yes/no)	1.099 (0.796–1.516)	0.567		
PNI (> 47.5/≤47.5)	0.534 (0.398–0.717)	< 0.001		
SII (> 588/≤588)	1.816 (1.334–2.471)	< 0.001	1.445 (1.046–1.997)	0.026
OPS				
1/0	1.865 (1.272–2.735)	0.001	1.938 (1.310–2.868)	0.001
2/0	3.043 (1.960–4.725)	< 0.001	2.757 (1.762–4.315)	< 0.001
3/0	5.744 (3.090–10.676)	< 0.001	4.779 (2.499–9.140)	< 0.001
GPS				
1/0	2.092 (1.507–2.906)	< 0.001		
2/0	3.435 (2.158–5.469)	< 0.001		
SIS				
1/0	1.637 (1.207–2.221)	0.002		
2/0	1.914 (1.084–3.379)	0.025		

*ESCC* Esophageal squamous cell carcinoma, *OPS* Osaka prognostic score, *GPS* Glasgow prognostic score, *CSS* Cancer-specific survival, *PNI* Prognostic nutritional index, *SII* Systemic immune-inflammation index, *SIS* Systemic inflammation score, *HR* Hazard ratio, *CI* Confidence interval, *TNM* Tumor node metastasis

### Development and validation of the nomogram

**Fig. 5:**
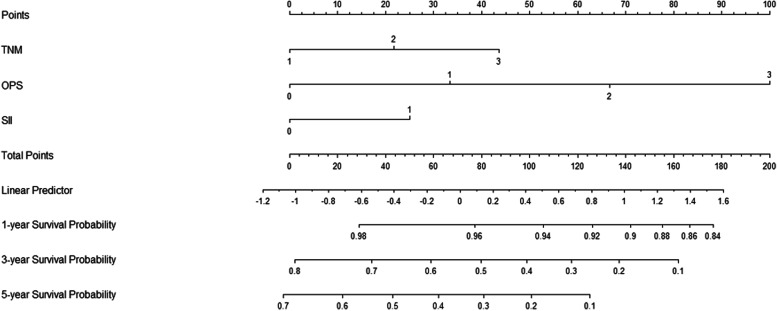
Nomogram established based on OPS. Nomogram based on OPS for predicting 1-, 3- and 5-year CSS in ESCC after radical resection

**Fig. 6:**
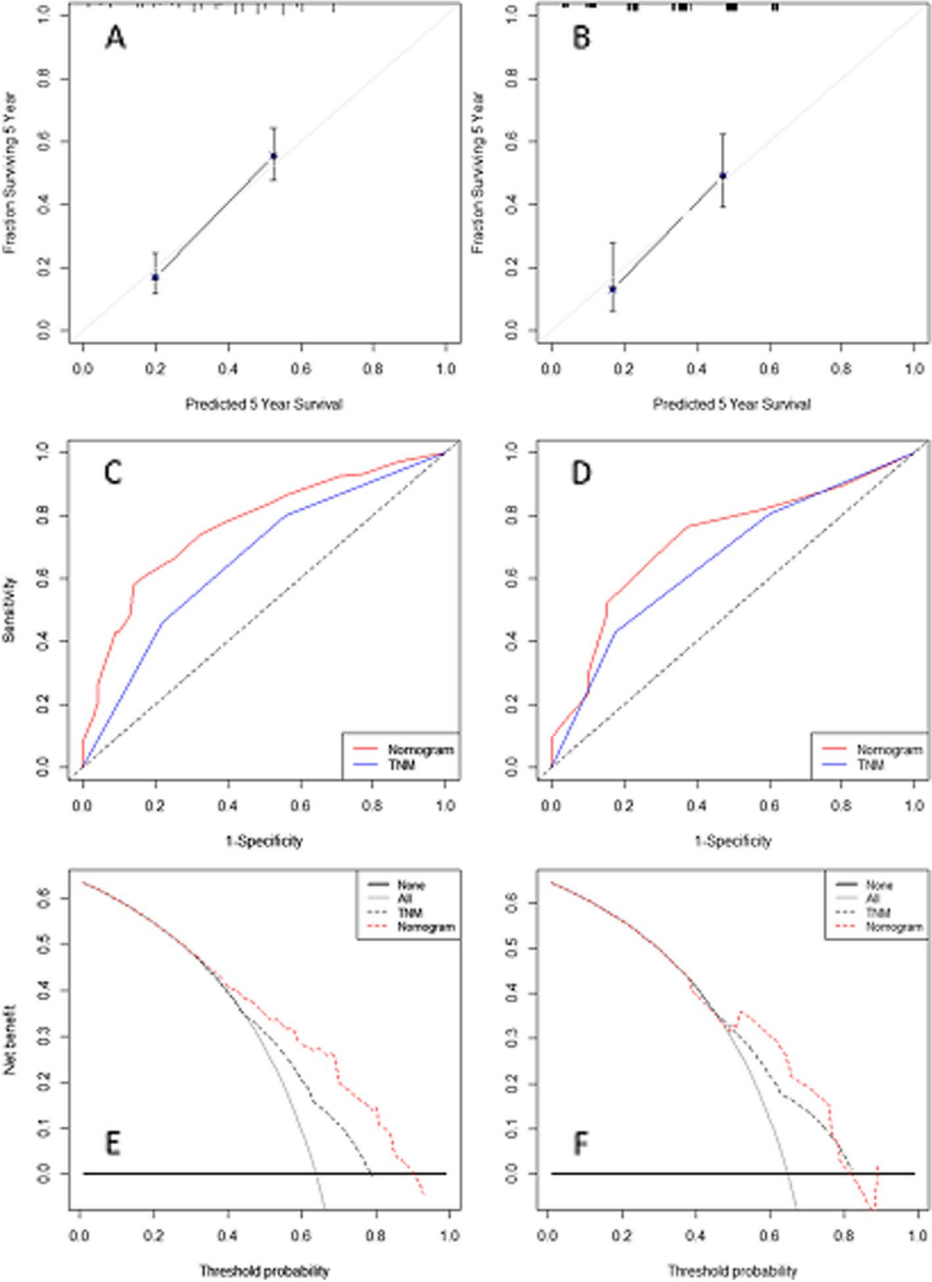
Nomogram validated. Calibration curves for 5-year survival prediction presented an acceptable agreement between two cohorts (**A-B**). Time-dependent ROC curve analyses revealed survival prediction was significant higher in nomogram than TNM stage (**C-D**). Decision curve analyses revealed nomogram model had higher overall net benefits than TNM stage (**E-F**)

Three variables according to the multivariate analyses (TNM, OPS and SII) were recruited to build a nomogram to predict individual survival (Fig. 5). The C-index was 0.68 in the training cohort and 0.67 in the validation cohort, respectively. An acceptable agreement between these two cohorts regarding the individual 5-year CSS prediction based on the calibration curves (Fig. 6A-B). The OPS-based nomogram had higher overall net benefits than TNM stages based on the time-dependent ROC analyses (Fig. 6C-D) and decision curve analyses (Fig. 6E-F). These results confirmed that the OPS-based nomogram can accurately and effectively predict survival in ESCC after radical resection.

## Discussion

To date, it is a dilemma to identify patients with ESCC who have aggressive behavior that leads to poor prognosis after surgical resection. Therefore, it is of great significance to explore more novel preoperative prognostic scores for postoperative prognosis in ESCC. The present study confirmed an integrative prognostic score of OPS, based on CRP, ALB and TLC, to predict clinical outcomes and prognosis in ESCC patients after radical resection. Compared with other indicators (GPS, SIS, PNI and SII), OPS had the largest AUC on the basis of ROC curves, which indicated that OPS had higher predictive ability on prognosis than other indicators. Then, OPS confirmed as a useful independent prognostic score. Moreover, a nomogram based on OPS was built and validated in the training and validation cohort, which can accurately and effectively predict prognosis in ESCC after radical resection.

Tumor prognosis is associated with inflammation and nutrition. CRP and ALB were the most widely recognized prognostic indicators in various cancers, including ESCC [6, 7]. GPS, based on CRP and ALB has been widely adopted as a systemic inflammatory and nutritional index, which indicated as a prognostic score not only for postoperative survival in ESCC but also for survival in various cancers [8–10]. In the current study, OPS, based on CRP, ALB and TLC, was performed to explore the clinical outcome in ESCC after radical resection. The results revealed that OPS had the higher abilities to predict prognosis than GPS (AUC = 0.683: 0.627, *P* = 0.0138) and confirmed as an independent score. Moreover, OPS in the present study also had the highest abilities to predict prognosis in ESCC compared with the other common indicators of SIS, PNI and SII in ROC analyses or Cox analyses.

The nutritional and/or inflammatory status may be influenced by a variety of non-cancer related conditions, which may lead to biased results. Therefore, more and more researchers are using these indicators in combination to reduce the potential bias and improve the prognostic value. Inflammation causes changes in tumor microenvironment and promotes proliferation, invasion and metastasis of cancer cells [23, 24]. Moreover, cancer itself may accelerate inflammation due to increased catabolism and malnutrition [25, 26]. There were two variables (CRP and ALB) in GPS and three variables (CRP, ALB and TLC) in OPS. CRP can induce a variety of inflammatory cytokines associated with cancers, such as interleukin-6 [27]. ALB, as a common marker regarding nutritional status, can activate a variety of cytokines, such as interleukin-1 and tumor necrosis factor-α [28]. When combined, GPS can effectively reflect potential inflammatory and nutritional status in tumor microenvironment. In addition, TLC plays an important role in the process of anti-tumor response, regulating angiogenesis, proliferation, apoptosis, and metastasis [29]. Therefore, the combination of additional TLC indicator resulted in a better stratification of OPS in prognosis than GPS in the current study and previous published study [15].

The present study explored an integrative prognostic score of OPS (based on CRP, ALB and TLC) to predict clinical outcomes and prognosis in ESCC after radical resection. Recently, a series of studies have revealed that nomogram is a better method to predict prognosis in various cancers [30, 31]. In the current study, our nomogram based on OPS containing three variables (OPS, TNM and SII) showed a better discrimination than the TNM staging system. The simply and easily obtained variables in nomogram, improves the application in clinical practice, allowing oncologists to use these nomogram to predict individual survival prediction in daily work. However, the prognostic value of OPS should be confirmed in more and more other cancers.

There are some limitations in the current study. First, this was a retrospective study. Second, this was a single-center study. Third, although the strict inclusion and exclusion criteria were adopted, levels of these serum variables may be affected by other conditions, therefore, the applications of OPS should be regard with caution. Forth, in order to better understand the prognostic value of OPS in patients with ESCC, we used two cohorts to verify the results. Although the prognostic value was validated, there was still a lack of additional independent external validation cohort. Therefore, the results of OPS may be correlated to certain bias and inaccuracy. Fifth, the prognostic value of OPS should be validated and confirmed in other more cancers. Sixth, endoscopic surgery (ES) and/or minimally invasive surgery (MIS) have become standard surgical methods for patients with EC in recent years. Compared with traditional surgical techniques, ES or MIS has a variety of advantages, such as decreased postoperative complications, shorter hospital stay and rapid recovery and discharge [32, 33]. The nutritional and immunological status for patients with ES or MIS has a lesser influence on the occurrence of postoperative complications. Accordingly, we should realize that the significance of OPS is likely to be reduced by the development of ES or MIS. Although the limitations existed, our developed OPS-based nomogram can accurately and effectively predict survival in ESCC after radical resection.

## Conclusion

The OPS is a novel, simple and effective index for prognosis in ESCC after radical resection. The nomogram based on OPS can accurately and effectively predict individual survival in ESCC after radical resection. The simply and easily obtained feature of OPS, improves the application in daily clinical practice. The OPS may allow for treatment stratification, thereby helping clinicians provide a more personalized approach to cancer treatment.

## Data Availability

The datasets used and/or analyzed during the current study are available from the corresponding author on reasonable request.

## Abbreviations

ESCC: Esophageal squamous cell carcinoma
SD: Standard deviation
CRP: C-reactive protein
ALB: Albumin
PLT: Platelet
TNC: Total neutrophil count
TLC: Total lymphocyte count
TMC: Total monocyte count
OPS: Osaka prognostic score
GPS: Glasgow prognostic score
SIS: Systemic inflammation score
TNM: Tumor node metastasis
PNI: Prognostic nutritional index
SII: Systemic immune-inflammation index
HR: Hazard ratio
CI: Confidence interval
